# Incidence of complications and functional outcomes following segmental femoral shaft fractures: a critical review of the literature

**DOI:** 10.1007/s00590-024-04065-8

**Published:** 2024-08-16

**Authors:** Anastasia Vasilopoulou, Spyridon Karampitianis, George D. Chloros, Peter V. Giannoudis

**Affiliations:** 1 Orthopaedic Surgery Working Group, Society for Junior Doctors, Athens, Greece; 2 Trauma and Orthopaedic Department, Korgialenio-Benakio Hellenic Red Cross Hospital, Athens, Greece; 3 Department of Surgery, Spital Walenstadt, Kantonsspital Graubünden, Spitalstrasse 5, 8880 Walenstadt, St Gallen, Switzerland; 4 Orthopaedic Surgery & Traumatology, University General Hospital “ATTIKON”, Medical School, National and Kapodistrian University of Athens, 12462 Athens, Greece; 5 Private Practice, Athens, Greece; 6 Academic Department of Trauma and Orthopaedics, School of Medicine, Clarendon Wing Leeds General Infirmary, University of Leeds, Floor D, Great George Street, Leeds, LS1 3EX UK; 7 NIHR Leeds Biomedical Research Center, Chapel Allerton Hospital, Leeds, UK

**Keywords:** Segmental femoral fractures, Trauma, Non-union, Outcomes, Complications

## Abstract

**Background:**

Although segmental femoral shaft fractures (SFSF) are very challenging to manage, there has been no critical evaluation of the current practices and outcomes. The aim of this study is to evaluate their characteristics, management trends, outcomes, and complications.

**Methods:**

A literature search was conducted via the SCOPUS, Embase (via SCOPUS) and MEDLINE (via PubMed) between 1995 and 2023. Studies were included if they reported patient demographics, mechanism of injury, classification of fractures, associated injuries, type of management, outcomes, and complications. Exclusion criteria: only diaphyseal fractures were included and proximal and distal femoral fractures were excluded from this study.

**Results:**

Overall, 22 studies met the inclusion criteria reporting on 313 patients. Mean age was 36.2 years with male–female ratio of 4.8 to 1. The majority were high-energy fractures secondary to road traffic accidents and 16% were open. The most commonly associated injuries included chest injury (27%) and lower leg fractures (24%). Treatment consisted of intramedullary nailing (IMN) (72%), plating (22%) or both combined (6%). Outcomes reported: good in 70%, fair in 10%, excellent in 19% and poor in 2% of cases. Mean time to union was 20 weeks. Complications are reported in 24% of cases, with most common delayed union (5%) and non-union (4%).

**Conclusion:**

SFSF are high-energy fractures occurring most commonly in young males, are open in 16% of cases and have significant associated injuries. In their overwhelming majority, IMN is the mainstay of treatment. The expected outcome is generally good in 70% of cases, although not devoid of complications in 24% of cases and patients must be aware of this during the consent process.

## Introduction

Femoral shaft fractures have an annual incidence ranging from 10 to 21 per 100,000 patients and are usually sustained either after high energy trauma in the young or low energy mechanism in the elderly with osteoporosis [1, 2]. Segmental femoral shaft fractures (SFSF) are defined as fractures occurring at two or more different levels of the femoral shaft [3] and are usually caused by high energy injuries, such as road traffic accidents (RTA), falls from height or heavy crush injuries [4].

Modern stabilisation techniques of SFSF include open reduction and internal fixation (ORIF) with plates and screws, external fixation, and intramedullary nail (IMN) [5], which remains the mainstay of treatment [4, 6]. However, SFSF have unique characteristics that make them challenging to manage effectively, as they are more prone to shortening, rotational deformity, malalignment, and additionally the compromised blood supply of the segments combined with the associated significant soft tissue insult puts them at higher risk of non-union [7–9]. To our knowledge, there has been no recent literature overview on SFSF, and therefore, the purpose of this study is to evaluate the current treatments and outcomes of SFSF in adults.

## Materials and methods

As a basis for this narrative review, a systematic search of the literature was conducted to assess the available evidence regarding SFSF in August 2023 using the 2009 Preferred Reporting Items for Systematic Review and Meta-analysis (PRISMA) guidelines [10].

All studies identified in the English language published onwards from 1995 via the following electronic databases were searched: Scopus, Embase (via Scopus) and MEDLINE (via PubMed). Specific research strings were formulated for each database using the following keywords and/or MeSH terms: 1) (Segment*)AND ( (femur)OR (femoral))AND (fractur*) and 2) Segment*AND fem* AND fractur*.

Inclusion criteria were studies presenting cases with SFSF reporting patient demographics, treatment methods and outcomes. Irrelevant studies, for example studies including Non-SFSF, segmental bone defects, studies including proximal and distal femoral fractures such as ipsilateral femoral neck and shaft fractures, femoral neck fractures, intercondylar fractures, intracapsular fractures were excluded. In addition, studies that do not report outcomes of treatment separately were also excluded. Moreover, biomechanical studies, in vitro studies, animal studies, review articles, non-English language literature, editorials, comments, opinions, letters to the editor, published abstracts, errata (unless they provide original data) were also excluded. The reference lists of the eligible studies and relevant review articles were cross-checked to identify additional relevant studies.

Two researchers (A.V and S.K) independently reviewed all studies (title, abstract and full text) that met inclusion criteria and extracted the relevant data. Any disagreements were resolved by the supervisor (G.C) through discussion.

### Data extraction

Data from the included studies were extracted in an Excel (Microsoft© Corporation) spreadsheet. Key variables included: patient demographics, mechanism of injury, fracture classification including open fractures, fracture site, associated injuries (orthopedic and non-orthopedic), type of treatment, complications, post-operative course including rehabilitation protocol, time to union, follow-up, and outcomes.

## Results

**Fig. 1:**
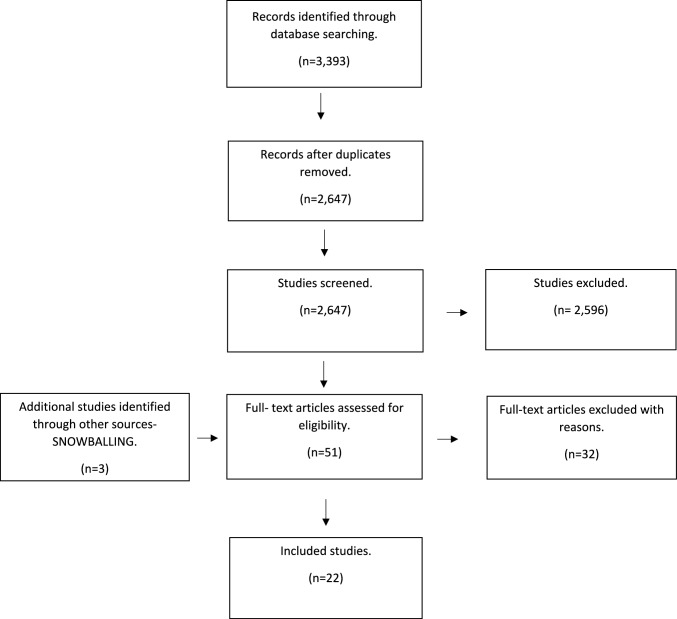
PRISMA flow diagram

The PRISMA flow diagram is shown in (Fig. 1). The search yielded 3,393 results and after duplicates were removed 2,647 articles were eligible for screening exclusion based on Title/Abstract. The full text of fifty-four references were assessed for eligibility. Thirty two studies were excluded at this stage: 2 duplicate studies [11, 12], 11 studies that reported mixed outcomes and there were no separate outcomes specific to SFSF [13–24], 7 studies that were in foreign language [23, 25–30], 7 studies that reported on SFSF with ipsilateral femoral neck fractures [20, 22, 24, 31–34] and 5 studies that were abstracts only [35–39]. Overall, 22 studies (18 retrospective case series [4, 7, 40–55]), 1 prospective control group study [56] and 3 case reports [57–59] with a total of 313 patients (314 fractures) met the inclusion criteria and formed the basis of this review.

### Demographics (Table 1)

**Table 1:** Eligible studies (chronological order)

Author	Patients (M/F)	Mean age (range)	Classification	Associated injuries
Chrisovitsinos (1997) [41]	1 (1/0)	38	OTA type C (1)	None
Wu/ (1997) [54]	56 (43/13)	37 (19–81)	ZONE 2,4:25	NR
			ZONE 2,3:10	
			ZONE 1,4:10	
			ZONE 1,3:3	
			ZONE 2,5: 3	
			ZONE 3,5: 1	
			ZONE 4,5: 1	
			ZONE 3,4: 3	
Deshmuk (1998) [42]	4 (3/1)	24 (16–36)	Winquist&Hansen 4 (4)	None
Broos (2002) [40]	3 (NR)	26 (NR)	Classified as segmental (SEINSHEIMER) (3)	NR
Kesemenli (2002) [48]	4 (4/0)	45 (17–67)	OTA type C (4)	NR
Mitchell (2010) [52]	10 (NR)	NA	OTA type C (10)	NR
Lee (2014) [51]	1 (1/0)	50 (NA)	OTA type C (1)	NR
Zheng/ (2014) [55]	15 (11/4)	29 (19–46)	OTA type C (15)	NR
Babaola (2016) [7]	8 (NA)	NA	OTA type C (8)	NA
Vaishya/ (2016) [58]	1 (0/1)	21 (NA)	NR	Chest injury (1)
				Pelvic fractures (1)
				Acetabular fractures (2)
Gannamani (2019) [45]	1 (1/0)	45	NR	None
Liu (2019) [4]	18 (14/4)	38 (27–52)	OTA type C (18)	Lower leg fractures (1)
				Chest injury (1)
				Pelvic fractures (2)
				Head Injury (1)
				Spine injury (1)
Hwang (2020) [57]	1 (1/0)	31	NR	Lower leg fractures (1)
				Upper limb fractures (1)
Jia (2020) [46]	1 (1/0)	34	OTA type C (1)	None
Layon (2020) [50]	1 (1/0)	28 (NA)	OTA type C (1)	NR
AnilBabu (2020) [43]	58 (N/R)	NR	OTA (58)	NR
Velmurugeasn (2020)[59]	2 (2/0)	42	NR	NR
Rollo (2020) [56]	40 (36/4)	40 (52–28)	Winquist&Hansen 3/4 (23/17)	Contralateral femoral injuries (7)
				Lower leg fractures (31)
				Upper limb fractures (16)
				Chest injury (40)
				Pelvic fractures (2)
				Acetabular fractures (4)
				Spine injury (11)
				Head Injury (19)
				Abdominal injury (28)
	Group 1: ORIF 20 (18/2)	40 (52–28)	Winquist&Hansen 3/4 (11/9)	Contralateral femoral injuries (4)
				Lower limb fractures (15)
				Upper limb fractures (8)
				Chest injury (19)
				Acetabulum fractures (2)
				Pelvic injury (1)
				Spine injury (6)
				Brain injury (9)
				Abdominal injury (14)
	Group 2: MIPO 20 (18/2)	40 (52–28)	Winquist&Hansen 3/4 (12/8)	Contralateral femoral injuries (3)
				Lower limb fractures (16)
				Upper Limb fractures (8)
				Chest injury (21)
				Acetabulum fractures (2)
				Pelvic injury (1)
				Spine injury (5)
				Brain injury (10)
				Abdominal injury (14)
Rkiba (2021) [53]	20 (NR)	33 (17–42)	OTA type C (20)	NR
Jitprapaikulsarn (2022) [12]	20 (17/3)	46 (22–66)	OTA type C (20)	Contralateral femoral injuries (1)
				Lower limb fractures (3)
				Upper limb fractures (4)
				Chest injury (1)
				Pelvic injury (1)
				Brain injury (1)
				Abdominal injury (2)
Ferreira (2022) [44]	10 (8/2)	35 (21–62)	OTA (10)	NA
Kook (2023) [49]	38 (33/5)	45 (18–74)	OTA type C (38)	NR

*NA* non-applicable, *MIPO* minimally invasive plate osteosynthesis

The demographics of the patients are shown in Table 1. There was a total of 313 patients (314 fractures) with 177 males and 37 females (M:F = 4.8) with a mean age of 36.2 years (range: 18–74 years). In 5 studies (99 patients), the sex could not be inferred as the demographics were mixed with nonsegmental fractures or were not reported [7, 40, 43, 52, 53].

### Mechanism of injury

Mechanism of injury is reported in 57% (178/314) of the fractures [4, 12, 40–42, 45, 46, 48–53, 55–59], whereas in the remaining is not reported [7, 43, 44, 53, 54]. The most common cause was RTA in 76% (135/178) [4, 12, 40–42, 45, 48–53, 55–59], followed by falls in 15% (26/178) [4, 46, 48, 49, 55, 56], work injury in 6% (11/178) [56], gunshot wound injuries in 1% (2/178) [48] and agricultural injury 1% (2/178) [56], ski injury 0.5% (1/178) [49] and crush injury 0.5% (1/178) [49].

### Classification (Table 1)

Fracture classification is reported in 98% (308/314) of fractures [4, 7, 12, 40–44, 46, 48–56] (Table 1). The AO/OTA classification [60] is used in 67% (205/308) of fractures [4, 7, 12, 41, 43, 44, 46, 48–53, 55], whereas in 14% (44/308) of fractures [42, 56], the Winquist and Hansen [61] classification was used. In 18% (56/308) of fractures, the femoral shaft was divided into 5 zones as described by Wu et al. [62] and the segments were classified accordingly. In the remaining 1% (3/308) of fractures the Seinsheimer classification [63] was used [40]. In 2% (6/314) of fractures no specific classification system was reported and the fractures were reported as “segmental” [45, 57–59]. Note that of the 314 fractures, 99% had one segment [4, 7, 40–46, 48–58] and 4 fractures were multisegmental [12, 55, 59]. Open fractures were 16% (50/314) [4, 41, 45, 54, 56], and according to the Gustilo-Anderson classification [64] 10% (5/50) were type I [4, 41], 44% (22/50) were type II [4, 56], 46% (23/50) were type III [41, 45, 56].

### Associated injuries (Tables 1, 2)

**Table 2:** Most common injuries associated with SFSFs

Associated injuries	Frequency
Chest injury [4, 12, 46, 48, 56, 58]	27% (42/153)
Lower leg fractures [4, 12, 56, 57]	24% (36/153)
Abdominal trauma [12, 56]	20% (30/153)
Head injury [4, 12, 48, 56]	14% (21/153)
Upper limb fractures [12, 56, 57]	14% (21/153)
Spinal injury [4, 56]	8% (12/153)
Acetabular fractures [56, 58]	4% (6/153)
Pelvic fractures [12, 48, 56]	4% (6/153)

*NR *not reported

Associated injuries are reported in 49% (153/313) [4, 7, 41, 42, 45, 46, 56–58] of the cases. Table 1 shows the associated injuries, per study, whereas Table 2, shows their cumulative frequencies. (Table 1).

The most common associated injury was chest injury with 27% (42/153) [4, 12, 46, 48, 56, 58], followed by lower leg injury with 24% (36/153) [4, 7, 12, 56, 57], abdominal trauma with 20% (30/153) [12, 56], head injury with 14% (21/153) [4, 7, 12, 48, 56] and upper limb fractures with 14% (21/153) [7, 12, 56, 57]. Spinal injuries [4, 7, 56] accounted for 8% (12/153), whereas acetabular [56, 58] and pelvic fractures [12, 48, 56] accounted for 4% (6/153) respectively each. (Table 2).

### Management and Outcomes (Table 3, 4)

**Table 3:** Follow-up, time to union, outcomes, and complications of SFSF eligible studies

Author	Mean Follow-up in years (range)	Mean time to Union in weeks (range)	Descriptive Outcomes	Complications
Chrisovitsinos (1997) [41]	1 (NA)	16	NR	Knee stiffness 1/1 (100%)
Wu (1997) [54]	2.6 (1.8–3.5)	23 (16–32)	Good (49) Fair (7)	Delayed union 12/56 (21%)
				Nonunion 7/56 (13%)
				Revision surgery 7/56 (13%)
Deshmuk (1998) [42]	NR	NR	NR	Rotational malalignment 2/4 (50%)
Broos (2002) [40]	NA	NR	NR	Delayed union 1/3 (33%)
				Revision surgery 1/3 (33%)
Kesemenli (2002) [48]	2 (1.7–2.8)	19 (12–20)	Good (2) Fair (2)	Knee stiffness 2/4 (50%)
Mitchell (2010) [52]	NA	48 (15–145)	Good (7) Poor (3)	Rotational malalignment 2/10 (20%)
				Knee stiffness 2/10 (20%)
				Delayed union 1/10 (10%)
				Revision surgery 1/10 (10%)
Lee (2014) [51]	NA	NR	Fair (1)	Delayed union 1/1 (100%)
				Revision surgery 1/1 (100%)
Zheng (2014) [55]	1 (0.5–1.5)	NR	Good (15)	Knee stiffness 2/15 (13%)
				Rotational malalignment 2/15 (13%)
Babalola (2016) [7]	0.9 (NA)	NR	Good (8)	Infection 1/8 (13%)
Vaishya (2016) [58]	0.9 (NA)	NR	Fair (2)	Nerve injury (2/2) (100%)
Gannamani (2019) [45]	1.9 (NA)	11 (NA)	Good (1)	None
Liu (2019) [4]	1.2 (1–1.9)	18 (NR)	Good (18)	Infection 2/18 (11%)
Hwang (2020) [57]	NR	NR	NR	Rotational malalignment 1/1 (100%)
Jia (2020) [46]	0.8 (NA)	NA	Good (1) Fair (1)	None
Layon (2020) [50]	NA	NR		Non-union 1/1 (100%)
				Revision surgeries 1/1 (100%)
AnilBabu (2020) [43]	NR	8 (NR)	NR	NR
Velmurugeasn (2020) [59]	1 (NA)	12 (NA)	Excellent (1) Fair (1)	Non-union 1/2 (50%)
				Revision surgery 1/2 (50%)
Rollo (2020) [56]	1.4 (0.9–3.7)	Total patients (40)	Good (37) Fair (3)	Revision surgery 3/40 (8%)
				Fat embolism 2/40 (5%)
				Hardware failure 3/40 (8%)
		Group1: ORIF 20 (14–23)	Good (20)	Fat embolism 1/20 (5%)
		Group 2: MIPO 20 (14–25)	Good (17) Fair (3)	Revision surgery 3/20 (15%)
				Fat embolism 1/20 (5%)
				Hardware failure 3/20 (15%)
Rkiba (2021) [53]	2.3 (0.9–4.4)	28 (NR)	Good (19) Poor (1)	Sural thrombophlebitis 1/20 (5%)
				Knee stiffness 3/20 (15%)
				Revision surgery 4/20 (20%)
Jitprapaikulsarn (2022) [12]	1.4 (1–3)	16 (12–20)	Excellent (13)	Limb shortening 4/20 (20%)
			Good (6)	Varus/valgus malalignment 6/20 (30%)
			Fair (1)	Hardware failure 1/20 (5%)
Ferreira (2022) [44]	0.75 (NA)	NR	Good (10)	None
Kook (2023) [49]	2.4 (1–5.6)	23 (13–39)	Excellent (32)	Non-union 6/38 (16%)
			Fair (6)	Revision surgery 5/38 (13%)

*NR* not reported, *NA* non-applicable

**Table 4:** Rank order list of frequency of complications

Complications	Frequency % (fractures)
Delayed union [40, 48, 51, 54]	5 (15/314)
Non-union [48–50, 54, 59]	4 (14/314)
Knee stiffness [41, 48, 53, 55]	2 (7/314)
Rotational malalignment [41, 42, 55, 57]	2 (7/314)
Varus/valgus malalignment [12]	2 (6/314)
Hardware failure [12, 56]	1 (4/314)
Limb shortening [12]	1 (4/314)
Infection [7, 48, 51, 54]	1 (3/314)
Fat embolism [56]	1 (2/314)
Nerve injury [58]	< 1 (1/314)
Thrombophlebitis [53]	< 1 (1/314)

Eighty-eight percent (88%) of fractures (278/314) were treated in a single stage [7, 12, 40–46, 49–54, 56, 59], whereas 12% (35/314) in two or more stages [4, 48, 55, 57–59]. In 1 study with < 1% (1/314) of fractures treatment staging is not reported [50]. Damage control orthopedics (DCO) is reported in 42% (131/314) [12, 43, 44, 56, 58, 59] of fractures, whereas in the remaining cases, it was either not reported or unclear. Seventy two percent of fractures (226/314) were treated with IMN [7, 40, 42–44, 46, 49, 50, 52–55, 57–59], 22% (68/314) ORIF with plate [12, 41, 45, 48, 51, 56], and in 6% (20/314) double fixation with both IMN and ORIF was achieved [4, 44].

IMN was performed with antegrade and retrograde reaming in 85% (209/246) of cases [4, 42–44, 49, 52, 54, 55, 58, 59], and in 5% (12/246) [7, 44, 49, 57] of cases respectively, whereas in 1% (3/246) were unreamed [40]. In 9% (22/246) of cases the authors do not report the procedure in detail [46, 50, 53].

Closed reduction was performed in 68% (213/314) of fractures [4, 7, 40–43, 46, 48–50, 53–55, 57, 59], while in 28% (89/314) open reduction was required [4, 12, 45, 52, 56]. The reduction method is not reported in a small minority of fractures, 4% (12/314) [44, 58]. No study reports incidence of infection and/or nonunion of open versus closed reduction.

The Rehabilitation protocol is reported in 79% (248/314) of fractures [7, 12, 40, 41, 43, 45, 48, 49, 54–56, 58, 59]. The majority of patients were instructed to be initially non-/partial-weightbearing and subsequently transitioned to full weightbearing based on the amount of callus presence. Specifically, in 29% of fractures (71/248) [7, 12, 45, 56, 58], partial weightbearing was initiated after 6 weeks, in 1% of fractures (2/248) [59] after 4 weeks, while in 48% (119/248) [41, 43, 48, 54] partial weightbearing was allowed when sufficient callus formation was identified on postoperative imaging. In 22% (56/248) [40, 49, 55] the transition from non-weightbearing to full weightbearing is not clearly mentioned. Mean follow-up was 1.43 years (range: 0.5–5.6 years) [4, 7, 12, 41, 43–46, 48, 49, 53–56, 58, 59].

Descriptive outcomes, without specific outcome instruments, are reported in 17 out of 22 studies [4, 7, 12, 44–46, 48–56, 58, 59]. while in 2 studies specific outcome instruments were used to evaluate the outcomes [56]. Overall, excellent outcomes are reported in 19% of fractures (46/247) [12, 49, 59], good outcomes are reported in 70% of fractures (173/247) [4, 7, 12, 44–46, 48, 52–56], fair in 10% (24/247) [12, 48–51, 54, 56, 58, 59] and poor in 2% (4/247) [50, 51, 53].

In 1 prospective study specific outcome instruments including Visual Analogue Scale (VAS) score, Harris Hip Score (HHS) and Knee Society Score (KSS) were used to evaluate the two patient groups treated with either plate fixation MIPO or ORIF with bone graft, separately during follow up period, showing that ORIF provided better results compared to MIPO [56]. In another study the Thoresen scoring system [12] was used to assess malalignment and post-operative range of motion, with excellent values in 65% of cases [47].

In 68% (213/314) of the fractures, union time is reported with a mean time to union of 20 weeks (range, 11–156) [4, 12, 41, 43, 45, 48, 49, 52–56, 59].

Complications are reported in 24% (75/314) of fractures [4, 7, 12, 40–42, 45, 46, 48–59] and are outlined in Table 3 (per study) and Table 4 (overall frequency). Revision surgery was performed in 8% (24/314) of fractures [40, 49–54, 56, 59], to address non-union [40, 49–54, 59] and hardware failure [56].

## Discussion

Non-SFSF are relatively infrequent injuries with an incidence of about 10/10,000 patients [65–67]. Only 2% of these are open fractures [68]. In general, these fracture types demonstrate an age and gender related bimodal distribution, as they result from high energy trauma in young male patients or fall from standing in elderly females [68, 69]. Associated injuries are common and often necessitate DCO [70]. Several treatment options exist with IMN being the gold standard [4, 6]. In this narrative review the authors are dealing with segmental femoral shaft fractures and not segmental femoral fractures which is a more generic term and may include proximal or distal femoral fractures.

In SFSF, the authors found that the mean age was 36.2 years (range, 18–74 years) and the majority were males (4.8 ratio). These findings show that the male preponderance is higher, and the age is lower in SFSF compared to Non-SFSF where the mean age was 68 years old and male to female ratio was 1:2 respectively [67]. Additionally, the most common mechanism in SFSF was by far an RTA in 76% of cases, with only 15% of cases being a fall, compared to 48% and 37% in the non-SFF counterpart respectively [1]. It can be concluded that SFSF are more frequently the result of high-energy injuries, and occur mostly in young males, compared to the bimodal distribution in Non-SFSF.

In this study the most common associated injuries include the chest (27%), lower leg (24%) and abdomen (20%). In a review of 26,357 non-SFSF fractures, the respective percentages were 18.9%, 20.5% and 6.2% [71]. It can be concluded that SFSF have significantly higher incidence of associated injuries, which would also be expected by the higher energy involved. However, SFSF associated injuries were reported in detail in 75% of cases and in another 33% of studies, associated injuries were not reported separately but collectively as “polytraumas”, which may or may not account for the discrepancy.

As far as classification, like in Non-SFSF, most SFSF studies (67%) report the AO/OTA classification [4, 7, 12, 41, 43, 44, 46, 48–53, 55]. Two studies [42, 56] report the Winquist and Hansen Classification [61], whereas one study [40] used the Seinsheimer classification [63]. In 1 study [54] the authors used a less known classification system, which divided the femoral shaft in 5 zones and described the fracture segments accordingly. Of note, one study used the AO/OTA classification for each segment separately [44]. Interestingly, only 4 fractures out of 314 had more than one segment [12, 55, 59], whereas the rest had one segment. Regarding open fractures according to Gustilo-Anderson classification, the findings in SFSF indicate a 16% of open fractures, which contrasts with Non-SFSF of 9% only [1], and the authors speculate that this may be because of the higher energy required to produce a SFSF.

Associated injuries often occur in conjunction with femoral shaft fracture, in both Non-SFF and SFF. Recent multicentre studies show that the most commonly associated orthopaedic injuries for Non-SFSF are lower leg fractures (20%), chest injuries (19%) and head injuries (14%) [68, 70, 72]. In this study, the majority of associated injuries included chest trauma (27%), followed by lower leg fractures (24%) and abdominal trauma (20%). Head injury incidences were comparable among SFSF and Non-SFSF. It can be concluded that SFSF present with higher rates of associated injuries compared to Non-SFSF, probably due to the more frequent polytrauma/higher energy mechanism. The authors tried to assess whether the treatment approach changes in patients with SFSF according to the nature and presence of associated injuries, however no specific pattern was identified.

Antegrade reamed IMN remains the gold standard procedure for Non-SFSF [68].This study found that 72% of fractures were treated with IMN [7, 40, 42–44, 46, 49, 50, 52–55, 57–59], 22% ORIF with plate [12, 41, 45, 48, 51, 56], and in 6% both IMN and ORIF were simultaneously performed [4, 44]. This combo approach was only reported by only two studies: Liu et al.[4] used it in a relatively young population (mean age 38 years, range 27–52). The authors used a plate first in order to maintain reduction and prevent rotational instability prior to reaming and inserting an antegrade IMN. Although the plate served the aforementioned purpose, it was arbitrarily left in situ, although they acknowledge that it could have also been removed [4]. Ferreira et al. [44] decided to use this combo approach in two middle aged patients whose segmental injury involved a relatively more distal segment.

As described in the Non-SFSF literature, ORIF does not provide better outcomes compared to IMN, ORIF can be used in diaphyseal fractures with further proximal or distal extension, where IMN may be contraindicated or infeasible [66, 73–75]. In the results for SFSF reported herein, the majority (85%) were treated with antegrade nails, and 99% of all nails were reamed. Although, in a recent meta-analysis, reamed or unreamed technique for femoral shaft fracture IMN remains controversial [28], reamed IMN is generally preferred due to shorter union time and lower rates of non-union as it was not found to increase blood loss and ARDS rates [28, 73]. However, the unreamed technique has the advantage of reducing operative time and therefore may still be indicated for patients with comorbidities, pathological femoral fractures, or severely injured patients [76, 77]. In 28% of SFSF open reduction was used to restore length, alignment, and rotation. Apart from these reasons, open reduction in SFSF can be used to stabilize the segmental part during reaming and therefore prevent devitalization of the fragment. This can be achieved by applying pressure downwards using a Hohmann retractor, by applying a Schanz pin to manipulate the fragment or by using a blocking screw [49]. In the Non-SFSF literature there were cases where open reduction rates were lower (33%), compared to SFSF [78]. However, in a recent study with subtrochanteric fractures with diaphyseal extension open reduction rates were significantly higher (48%) [79]. The reason for this could be that passing the guide wire becomes easier especially in more complex fracture patterns and there was no difference between union rates in closed and open reduction method [78, 80].

As far as rehabilitation, the majority of patients were advised initial non-weightbearing and progress to full weightbearing based on the amount of callus present. In 29% of SFSF partial weightbearing was initiated after 6 weeks post-operatively, and in 48% advancement was based on callus formation, on a case-by-case basis. On the other hand, in Non-SFSF treated with IMN, partial weightbearing is initiated the first two weeks and is followed by full weightbearing after 4 weeks according to the callus presence [81, 82].

In SFSF the functional outcomes reported are generally good in 70% of cases, compared to the literature for the Non-SFSF (93%) [73], whereas in 19% are excellent. In addition, in SFSF mean time to union is 20 weeks, which is significantly longer in comparison with Non-SFSF which is 14 weeks [5, 73, 74, 83, 84]. The authors speculate that because SFSF are more often the result of high-energy injury compared to the Non-SFSF as alluded to earlier, this may cause a higher disruption in fracture biology due to the nature of the injury and therefore compromised vascular supply and slower healing times.

The most common complications reported in SFSF include delayed union (5%) followed by non-union (4%), knee stiffness (2%), and rotational malalignment (2%). These occurred less frequently in Non-SFSF in which delayed union and non-union were 2.2% and 2.1% respectively [73]. Of interest, SFSF studies do not report the amount of shortening except for 1 study [12] which would be interesting to know as an outcome of treatment of SFSF.

## Limitations

This study has some limitations. The level of evidence is low, as most studies are retrospective case series and case reports. The mean follow-up was 1.45 years (range: 0.75–3 years), which is short and even absent in some studies. None of the studies report comorbidities, American society of anesthesiology grade (ASA) or injury severity score (ISS) during admission of the cases included. In addition, in 33% of cases [52, 54] were described as “polytraumas” which is a general term, and the associated injuries were not all recorded in detail. Only 7 studies report DCO [12, 43, 44, 49, 56, 58, 59], while in the remaining ones, even the newer ones, it is not reported whether or how it was done. Except for 2 studies [12, 56], all studies reported descriptive outcomes and do not employ specific outcome instruments. Furthermore, none of the included studies report mortality rates. Only in 2 studies mean operative time is reported [12, 47]. In addition, time to union is not reported in detail in many studies or the results were mixed with non-segmental fracture types and the exact union time could not be estimated [7, 40, 42, 44, 50, 51, 55, 57, 58]. There were also studies where the outcome of the patients was partially reported [40–43, 57].

## Conclusion and future directions

SFF are high-energy complex injuries which are challenging to manage. Their mean age is significantly younger than Non-SFF, with a higher male preponderance and a higher associated injury incidence. They are open in about a quarter of cases, which is significantly higher than Non-SFF. Like Non-SFF, IMN is the mainstay of treatment, but SFF have about 50% longer time to union and about double the rates of nonunion compared to Non-SFF.

Based on these limitations, future research should aim in eliminating the aforementioned limitations: higher quality studies should include a detailed description of the patient group, including comorbidities, ASA, ISS, associated trauma injuries, DCO versus early total care, specific outcome instruments, mortality rates, follow-up time and time to union. In addition, studies comparing SFSF vs NSFSF groups are needed to directly assess the differences and provide sturdier insights, which are currently lacking.
